# Maternal and Fetal SERPINA3 Polymorphisms and Risk of Preeclampsia: A Dyad and Triad Based Case-Control Study

**DOI:** 10.3390/cimb47110952

**Published:** 2025-11-17

**Authors:** Hsi-Hsuan Yang, Claire Baldauf, Trevor A. Pickering, Håkon K. Gjessing, Sue Ann Ingles, Melissa Lee Wilson

**Affiliations:** 1 Department of Population and Public Health Sciences, Keck School of Medicine, University of Southern California, Los Angeles, CA 90033, USA; hsihsuan@usc.edu (H.-H.Y.);; 2 Department of Pediatrics, Keck School of Medicine, University of Southern California, Los Angeles, CA 90033, USA; 3 Centre for Fertility and Health, Norwegian Institute of Public Health, 0379 Oslo, Norway; 4 Department of Global Public Health and Primary Care, University of Bergen, 5009 Bergen, Norway

**Keywords:** preeclampsia, HELLP syndrome, polymorphism, genetics, parent-of-origin

## Abstract

Serine protease inhibitor A3 (SERPINA3), also called α-1-antichymotrypsin, is a serine protease involved in placental dysfunction. This study examines *SERPINA3* polymorphisms and haplotypes for associations with maternal hypertensive disorders of pregnancy (HDPs) and preeclampsia with severe features (sPE) or Hemolysis, Elevated Liver Enzymes, and Low Platelet (HELLP) syndrome in mother–baby dyads (HDP) and mother–father–baby triads (sPE/HELLP). This retrospective case–control study examined two patient cohorts, HDPs and severe PE/HELLP syndrome. The HDP population included cases (*n* = 142) and controls (*n* = 168) of mother–baby dyads recruited from a large, urban, safety-net hospital in Los Angeles. The sPE/HELLP syndrome population included cases (*n* = 189) and controls (*n* = 28) of mother–father–baby triads recruited through HELLP syndrome research websites. Cases were verified by medical chart abstraction when possible. Two *SERPINA3* SNPs, rs4934 and rs1884082, were genotyped from saliva samples, mouthwash, or buccal swabs. The Haplin package in R was used to perform genetic association analyses. No evidence of increased risk related to individual *SERPINA3* SNPs or haplotypes for the developing HDPs or sPE/HELLP was found in individual nor combined cohorts. In the HDP cohort, the g-a haplotype (relative to T-G haplotype) was borderline significant for increased risk of HDPs when carried by the child (double dose: RR = 1.58, 95% CI: (1.00, 2.52), *p* = 0.05). We observed significant parent-of-origin (PoO) effects in the combined cohort: specifically, an increased risk of HDPs/sPE/HELLP if the mother carries a double copy for both rs4934 (RR = 3.03, 95% CI (1.50, 6.09), *p* < 0.01) and rs1884082 (RR = 2.38, 95% CI (1.22, 4.71), *p* = 0.01). A reduced risk of HDPs/sPE/HELLP was observed for rs4934 (RR = 0.54, 95% CI (0.31, 0.98), *p* = 0.04) and rs1884082 (RR = 0.52, 95% CI (0.30, 0.91), *p* = 0.02) with child carriage of the maternally inherited allele. In contrast, child carriage of a paternally inherited copy of the variant allele for rs4934 increased risk of HDPs/sPE/HELLP (RR = 1.54, 95% CI (1.09, 2.20), *p* = 0.02). There was no evidence that *SERPINA3* gene polymorphisms and haplotypes were associated with risk of HDPs or sPE/HELLP. However, significant PoO effects were observed in the combined cohort analysis, with child carriage of rs4934 that is maternally inherited decreasing HDPs/sPE/HELLP risk while a paternally inherited copy increases risk, suggesting a role for maternal–fetal genomic incompatibility.

## 1. Introduction

Hypertensive disorders of pregnancy (HDPs) complicate 6% to 8% of all pregnancies [1]. HDPs are among the major contributors to adverse maternal health outcomes in the United States [2]. HDPs vary in severity and include preeclampsia, eclampsia, chronic hypertension with superimposed preeclampsia, and gestational hypertension, as defined by the American College of Obstetricians and Gynecologists (ACOG) [3]. Hemolysis, Elevated Liver Enzymes, and Low Platelet (HELLP) syndrome is considered to be a severe complication of HDPs [4], though there may be some variability in risk factors between HDPs and HELLP syndrome [4]. These conditions can have significant short- and long-term complications for both maternal and child health. Short-term maternal complications include heart failure, hemorrhage from placental abruption, stroke, renal dysfunction, and pulmonary edema [5,6]. Long-term postpartum complications include increased risk of cardiovascular events, stroke, chronic kidney disease, and type 2 diabetes [7,8]. Fetal and neonatal complications include fetal growth restriction and preterm birth, while long-term effects include poorer neurodevelopmental outcomes and a higher risk of metabolic disorders and cardiovascular disease in adulthood [9,10,11,12].

Preeclampsia (PE) is characterized by new-onset hypertension (≥140 systolic or ≥90 diastolic/mmHg) and proteinuria (>300 mg/24 h) or other signs of organ dysfunction (e.g., elevated liver enzymes) occurring after 20 weeks of pregnancy [13].

While the public health significance of PE is well recognized, its molecular pathogenesis has yet to be completely understood. PE is known to be associated with changes in expression of placental genes; however, the specific patterns of risk-related gene expression in PE are incompletely elucidated, highlighting the need for further research into the underlying molecular mechanisms [14]. Serine protease inhibitors (SERPINs) such as SERPINA3, A5, A8, B2, B5, andB7 are among the placentally expressed genes associated with PE [15]. Among the SERPINs, SERPINA3 has drawn particular attention due to its potential role in placental dysfunction. SERPINA3, also called α-1-antichymotrypsin, is involved in a wide range of biological processes including inflammation. Maternal tolerance of the semi-allogenic fetus is tightly regulated in pregnancy. SERPINA3 helps regulate the maternal inflammatory response by binding to serine proteases and undergoing a conformational change that prevents proteolytic activity [16,17]. As reviewed by Chelbi et al., SERPINA3 is up-regulated in human placental diseases in association with a hypomethylation of the 5′ region of the gene [18]. A study by Auer et al. found that protein levels of SERPINA3 are higher in serum of pregnant women affected by PE and fetal growth restriction [19]. These findings indicate that SERPINA3 is both differentially expressed and epigenetically dysregulated in human placental diseases, implicating its potential role as a marker of placental dysfunction. Further, Yang et al. identified SERPINA3 as one of the genes upregulated in PE [20]. It also showed that SERPINA3 positively correlated with plasma cells and eosinophils, but negatively with anti-inflammatory cells like macrophages and neutrophils. The correlation pattern indicates that elevated SERPINA3 expression may contribute to an inflammatory environment in the placenta.

In addition to placental diseases, SERPINA3 has been found to be associated with various human diseases, such as Alzheimer’s disease [21] and chronic obstructive pulmonary disease [22], and multiple forms of cancer, including glioblastoma, colorectal cancer, endometrial cancer, breast cancer, and melanoma [23].

Our study adds to the current knowledge of SERPINA3 in several ways. First, few studies have explored *SERPINA3* polymorphisms in relation to PE risk, especially for the Hispanic population. Second, we explored mother–child dyads and mother–father–child triads to provide a more comprehensive understanding of genetic contributions to PE risk, including parent-of-origin (PoO) effects. Lastly, the existing studies on *SERPINA3* polymorphisms related to pregnancy complications are limited; our study helps to add and expand upon the current knowledge of SERPINA3’s role in HDPs.

The main objective of this study is to investigate the relationship between two *SERPINA3* alleles and HDP/PE with severe features (sPE)/HELLP syndrome.

## 2. Materials and Methods

### 2.1. Subjects

HDPs: This retrospective case–control study included mother–baby dyads, of which 142 were diagnosed with HDPs and 168 had normotensive pregnancies. Participants were recruited from a large, urban, safety-net hospital in Los Angeles. Participants were identified via delivery logs between 1999 and 2006 (*n* = 105, 33.8%) and during their postpartum hospital stay from 2007 to 2008 (*n* = 206, 66.2%). Cases were women diagnosed with eclampsia, PE (including superimposed PE), gestational hypertension, or HELLP syndrome, and were verified through chart review. Controls were women from the same population and time period with no clinical diagnosis of HDPs during pregnancy.

Severe PE/HELLP: This retrospective internet-based cohort includes mother–father–baby triads (*n* = 217). Participants were recruited through two HELLP syndrome-centered websites (www.hellpsyndromesociety.org or https://www.facebook.com/pages/Hellp-Syndrome-Research-at-USC/163745723652843, accessed on 5 November 2025). The case–cohort (*n* = 189) consisted of women with self-identified HELLP syndrome, with verification through medical record review for 66.1% of cases. Some participants in the case–cohort recruited friends who gave birth within five years of the affected pregnancy and reported no HDP history during pregnancy for the control cohort (*n* = 28 triads).

### 2.2. Case Definition

HDP: PE cases were identified if the participants had a systolic blood pressure ≥140 mmHg and/or a diastolic blood pressure ≥90 mmHg on two measurements taken at least 6 h apart, accompanied by proteinuria, as defined by a dipstick reading of ≥1 or a total of ≥300 mg/dL in a 24 h urine sample. Participants with high blood pressure without proteinuria were classified as having gestational hypertension. As there was no significant difference observed between these groups, they were combined for analysis.

Severe PE/HELLP: Self-reported HELLP cases were confirmed based on the following criteria: (1) hemolysis as indicated by either abnormal red blood cells on a peripheral blood smear or an LDH level of ≥600 IU/L, (2) liver enzyme elevation, defined as AST or ALT ≥ 70 U/L, and (3) platelet count < 100,000/µL, regardless of the presence of proteinuria or hypertension. Participants meeting at least two of these three criteria were categorized as having severe PE, as all exhibited hypertension (BP ≥ 160/110 mmHg on two separate readings taken at least 6 h apart) and proteinuria (≥500 mg/dL in a 24 h urine sample or a +3 dipstick reading on two occasions at least 6 h apart).

### 2.3. Questionnaire

A standardized risk questionnaire in English or Spanish was used for data collection. Information on personal and family medical history, reproductive and sexual history, obstetric history, and other risk factors was collected.

### 2.4. Chart Abstraction

For the HDP population, medical records were obtained from the delivery hospital for both cases and controls. For the severe PE/HELLP population, medical records were requested from the treating obstetrician and delivery hospital. Medical records were reviewed by one of the investigators (M.L.W.) to verify diagnoses. Data abstraction was conducted using a standardized data abstraction form, which included information about obstetric history, prenatal visits, delivery, and coexisting medical conditions.

### 2.5. Polymorphisms Selection

Two SNPs within the *SERPINA3* gene were selected for analysis to represent the majority of genetic variation within the gene: rs4934 and rs1884082. SNPs were selected if at least one of the following criteria was met: (1) recognized as a functional polymorphism in published studies, (2) related to health outcomes in the peer-reviewed literature, (3) located in a coding region and causing a non-synonymous amino acid change, or (4) found in a regulatory or upstream region. Information about the selected SNPs is shown in Table 1a–c. We repeated genotyping for 5% of all samples as a validation measure and found no discrepancies. The genotyping failure rate was 5.1% for rs1884082 and 7.7% for rs4934 in the cohorts.

### 2.6. Sample Collection

DNA samples were collected using buccal swabs (87%) or mouthwash (13%) for both mothers and infants in the HDP cohort. DNA samples of the severe PE/HELLP syndrome cohort were obtained via Oragene saliva kits (DNA Genotek, Ottawa, ON, Canada) (89%) or buccal swabs (11%). All mouthwash and saliva samples were extracted using ethanol precipitation. Buccal swabs were extracted using QIAmp DNA mini kits following the manufacturer’s protocol (Qiagen, Valencia, CA, USA). Genotyping failure rates were not affected by the DNA sample collection method.

### 2.7. Statistical Analysis

The analysis of the mother–baby dyads in the HDP cohort and mother–father–baby triads in PE/HELLP syndrome cohort was performed by using the Haplin package (Version 7.3.2) for R programming language (R Foundation for Statistical Computing, Vienna, Austria) [26]. Haplin is a software tool designed for genetic association analysis of case-parent triad data with multiple markers. It incorporates both complete and incomplete control triads and estimates relative risks (RRs), confidence intervals, and *p*-values associated with each variant/haplotype. Haplin also evaluates parent-of-origin (PoO) effects and uses maximum likelihood estimation to utilize data from dyads and triads with missing genotypic data [27]. It is statistically expressed as Relative Risk Ratio (RRR) = RR_M,j_/RR_F,j_, which is a measure of the relative risk of an allele A_j_ inherited from the mother as opposed to father [27]. Post hoc power calculations and free response models were also performed by Haplin [27]. All analyses were performed in R programming software (Version 4.4.2).

The analysis of continuous variables for maternal demographic and clinical characteristics is presented as means ± standard deviations or medians with interquartile ranges, while categorical variables are presented in counts and percentages. Demographic and clinical characteristics are described for both cohorts stratified by disease status.

Relative risks and 95% confidence intervals (CIs) for parent-of-origin effects, single- and double-dose effects, and haplotypes were evaluated using a two-sided significance level of α = 0.05. Due to sample size limitations and the pilot nature of this study, we opted not to adjust for multiple comparisons to minimize the likelihood of Type II errors.

We calculated the effect size we can detect with 142 case dyads and 168 control dyads, as well as with 189 case triads and 28 control triads. Assuming a minor allele frequency of 10% and a Type I error rate of 5%, we detected an OR of 1.5 for both lowest minor allele frequency (MAF) of 0.27 and highest MAF of 0.48 for the HDP cohort, and ORs of 1.7 and 1.6 at the same respective MAFs for the severe PE/HELLP cohort.

## 3. Results

### 3.1. Participants

**Figure 1:**
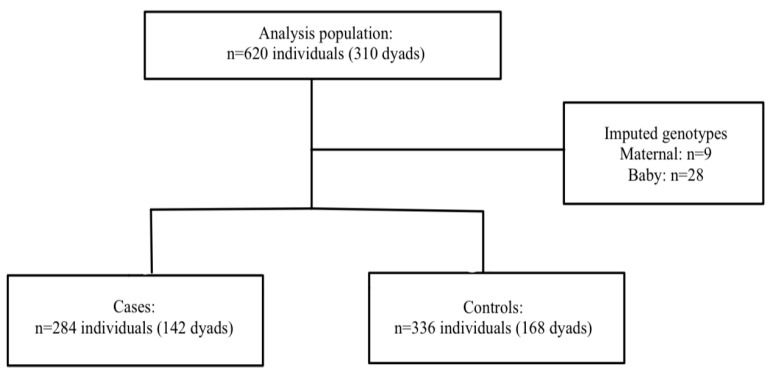
Participant flow chart in HDP population.

HDPs: A total of 620 mothers and babies (*n* = 310 dyads) were included in the final analysis. Nine mothers and 28 babies were missing at least one of the genotype data points. Haplin’s EM algorithm, implemented as part of the analysis, was used to impute the missing genotypes. Among the 142 cases, 68.3% were diagnosed with PE, 2.1% with superimposed PE, and 29.6% with gestational hypertension. The participant flow chart is shown in Figure 1.

**Figure 2:**
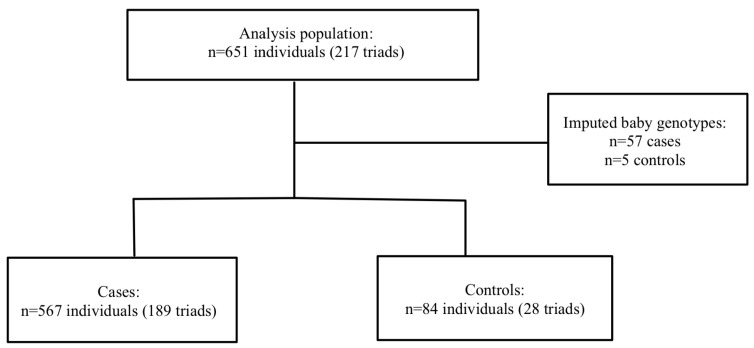
Participant flow in severe PE/HELLP syndrome population.

Severe PE/HELLP: A total of 217 mother–father–baby triads, 189 cases triads, and 29 control triads were included in the analysis. Haplin’s EM algorithm imputed the missing genotypes for 57 cases (10.1%) and 5 controls (5.7%). Within the sPE/HELLP cohort, 59.2% of the cases in the severe PE/HELLP syndrome population were classified as severe PE, and 40.8% were classified as HELLP syndrome. The participants flow chart is shown in Figure 2.

### 3.2. Maternal Demographics

HDPs: Table 2 shows the maternal demographics and clinical characteristics divided into HDP and severe PE/HELLP syndrome cohorts with corresponding case–control status. The HDP cohort included primarily women of Hispanic ancestry (95.8% for cases, 96.4% for controls). The cases exhibited lower gestational ages at birth (cases: 36.8 ± 3.4 and controls: 38.7 ± 2) and lower average birth weights (cases: 3060.0 g and controls: 3288.0 g) compared to controls. The nulliparity rate was higher among the cases (43% for cases; 30.7% for controls).

Severe PE/HELLP: All the participants in the severe PE/HELLP syndrome cohort were White. Cases exhibited lower gestational ages compared to controls (cases: 33.7 ± 3.8 and controls: 39.6 ± 1.8). In addition, the case–cohort had a lower average birth weight than the control cohort (cases: 2540.1 g, controls: 3242.3 g).

Table 2 shows the maternal demographics and clinical characteristics divided into HDP and severe PE/HELLP syndrome cohorts with corresponding case–control status.

### 3.3. Individual SNP Analysis

HDPs: There were no statistically significant associations between *SERPINA* SNPs and HDPs for either mother or child observed in the study cohort (Table 3a).

Severe PE/HELLP: No statistically significant associations between maternal and child *SERPINA3* polymorphisms and severe PE/HELLP syndrome were observed in this study cohort (Table 3b). Associations between father and child were assessed through imprinting status.

Combined: A combined analysis was performed due to the similarity of RR ratios in both the HDP and severe PE/HELLP syndrome cohort. The combined data of 527 triads were included in the analysis. For rs1884082, five triads were excluded from the original 527 triads due to Mendelian inconsistencies. For rs4934, four triads were removed due to Mendelian inconsistencies, and two triads were removed due to low frequencies. No statistically significant associations were observed in the combined cohort (Table 3c).

### 3.4. Haplotype Analysis

HDPs: In total, 16 of the 310 dyads were excluded due to low-frequency haplotypes, leaving 294 dyads for analysis. No statistically significant associations with risk of HDPs were found in either mother or child haplotypes (Table 4a).

Severe PE/HELLP: In total, 29 triads of the 217 triads were excluded from the analysis due to Mendelian inconsistencies (*n* = 7) and low-frequency haplotypes (*n* = 22). The remaining 188 case–control triads did not show any statistically significant relationship between maternal and child *SERPINA3* haplotypes and risk of severe PE/HELLP syndrome (Table 4b). A possible association was observed for the g-a haplotype (relative to T-G haplotype), suggestive of a possible protective effect when maternally carried (Single dose: RR = 0.69, 95% CI: (0.46, 1.05), *p* = 0.08).

Combined: A total of 45 dyads or triads were removed from the analysis due to Mendelian inconsistencies (*n* = 7) and low-frequency haplotypes (*n* = 38). No statistically significant associations were observed between the g-a haplotype (relative to T-G haplotype) and HDPs in the combined cohort for maternal alleles, but associations were borderline significant for child alleles (Double dose: RR = 1.58, 95% CI: (1.00, 2.52), *p* = 0.05) (Table 4c).

### 3.5. Parent-of-Origin Analysis

No PoO effects were observed in either HDP or sPE/HELLP cohorts (Table 5). However, in the combined cohort, we found statistically significant PoO effects. First, we observed an increased risk of HDPs if the mother carries a double copy of both rs4934 (RR = 3.03, 95% CI (1.50, 6.09), *p* < 0.01) and rs1884082 (RR = 2.38, 95% CI (1.22, 4.71), *p* = 0.01). We also observed a decreased risk of HDPs with rs4934 (RR = 0.54, 95% CI (0.31, 0.98), *p*= 0.04) and rs1884082 (RR = 0.52, 95% CI (0.30, 0.91), *p* = 0.02), associated with child carriage of the maternal copy. Conversely, child carriage of the paternal copy of rs4934 is associated with an increased risk of HDPs (RR = 1.54, 95% CI (1.09, 2.20), *p* = 0.02).

**Table 5:** Combined parent-of-origin analysis of associations between maternal and child *SERPINA3* haplotypes and risk of HDPs and severe PE/HELLP syndrome.

SNP	Allele	Allele Frequency (%)	Maternal				Child							
			Single-Dose RR (95% CI)	*p*	Double-Dose RR (95% CI)	*p*	Single- Mat Dose RR (95% CI)	*p*	Single- Pat Dose RR (95% CI)	*p*	Double-Dose RR (95% CI)	*p*	Ratio m-p RRR (95% CI)	*p*
rs4934	A	30.4	1.19 (0.83, 1.72)	0.35	3.03 (1.50, 6.09)	<0.01	0.54 (0.31, 0.98)	0.04	1.54 (1.09, 2.20)	0.02	1.13 (0.69, 1.88)	0.63	0.35 (0.18, 0.71)	<0.01
rs1884082	G	36.4	1.21 (0.85, 1.72)	0.29	2.38 (1.22, 4.71)	0.01	0.52 (0.30, 0.91)	0.02	1.38 (0.98, 1.94)	0.07	1.09 (0.68, 1.75)	0.72	0.38 (0.19, 0.74)	<0.01

HWE *p*-values: rs4924 (*p* = 0.01), rs1884082 (*p* < 0.01). Note: RRR = RRM/RRF.

## 4. Discussion

This study analyzed maternal and child polymorphisms and haplotypes in the *SERPINA3* gene for associations with HDPs and severe PE/HELLP syndrome. No evidence of statistically significant risk of HDPs or severe PE/HELLP was observed in either cohort, nor in the combined cohort. While no significant PoO effects were detected in the HDP or severe PE/HELLP cohorts individually, the combined cohort analysis was significant for both rs1884082 and rs4934. Specifically, we observed (1) an increased risk of HDPs/sPE/HELLP if the mother carried a double copy of the variant allele, (2) a decreased risk of HDPs/sPE/HELLP if the child carried the maternally inherited copy, and (3) an increased risk of HDPs/sPE/HELLP if the child carried the paternally inherited copy. The statistically significant RRR suggests that child carriage of a maternal copy has a protective effect compared to the paternal copy, suggesting a potential imprinting effect. These findings indicate that PoO effects play a role in predisposing pregnant women to HDPs and HELLP syndrome. Nevertheless, further studies are needed to confirm these observations.

Our analysis on individual SNPs did not find a statistically significant association between rs1884082 and rs4934 polymorphisms and HDPs, severe PE/HELLP syndrome, or the combined cohort. However, a study by Chelbi et al. found that the T allele of the rs1884082 SNP was a potential risk factor IUGR, while the rs4934 SNP was related to an increased risk of PE, particularly in the Hispanic population [18]. Our combined PoO analysis suggests that the source of the inherited risk allele—whether maternal or paternal—contributes to HDP risk, implicating possible maternal–fetal genotypic mismatch.

Our findings suggest that maternal–fetal genotype mismatches at the *SERPINA3* locus may increase the risk of HDPs. This is consistent with a study by Sinsheimer et al., in which maternal–fetal genomic incompatibility at immune loci predisposed individuals to Rh incompatibility. Specifically, when the mother is homozygous for a null allele, and the baby inherits an antigen-coding allele from the father, it triggers a harmful maternal immune response [28]. Similarly, maternal–fetal genomic incompatibility at immunological loci has been described as a risk factor for subsequent schizophrenia [29]. These results support the idea that maternal–fetal genomic interactions likely contribute to pregnancy outcomes.

The lack of statistically significant associations for individual SNPs and haplotypes may reflect relatively small sample sizes. Nonetheless, we cannot exclude the possibility that a mixture of maternally and paternally inherited alleles obscured any associations due to parental imprinting. In support of this possibility, we observed that, among the combined cohorts, both rs1884082 and rs4934 demonstrated PoO-specific effects. A single copy of the maternally inherited allele is associated with a reduction in HDP risk, whereas a single copy of the paternally inherited allele increases HDP risk. A parent-of-origin effect was defined by the interaction effect of maternally derived and paternally derived alleles [27]. The underlying complexity of HDPs may obscure the relationship between these polymorphisms and PR risk.

SERPINA3 is a protease inhibitor and is a specific inhibitor of elastase, which belongs to the matrix metalloprotease family and plays an essential role during the implantation process [30]. Such inhibitors also serve as storage proteins (ovalbumin), carrier proteins (steroid-binding globulin), and hormone precursors (angiotensinogen or SERPINA8) without inhibitory function. SERPINs are involved in the maintenance of homeostasis and regulate several molecular pathways, such as inflammation, coagulation, fibrinolysis, complement activation, and phagocytosis. All of these cascades are affected in placental diseases [31,32]. Taken together, these findings may suggest that an increased SERPINA3 synthesis may inhibit the matrix disintegration necessary for efficient implantation [15]. These findings are consistent with our understanding of the pathogenesis of PE and may suggest that SERPINA3 may have an early role in predisposing to placental diseases.

The functional impact of the rs4934 polymorphism is unclear. It may play a role in reducing protein secretion, altering protein expression or function, or acting through linkage disequilibrium (LD) with other functional variants. rs1884082, which is in strong LD with rs4934, influences transcription factor binding and is associated with significantly higher serum alpha-1-antichymotrypsin (ACT) levels. It also increases inflammatory responses and has been previously linked to PE.

There are several limitations in this study that should be considered. First, both rs1884082 and rs4934 SNPs in the combined analysis show deviation from Hardy–Weinberg Equilibrium (HWE). This could be due to various reasons including, but not limited to, genotyping error, maternal–fetal genotypic interaction, selection bias, and population stratification [33,34,35]. This study was limited by the scarcity of control families in the HELLP/severe disease cohort resulting from our recruitment method. However, the analytic methods we used allow for assessment of case-only studies [36]. Therefore, having few control families did not substantively alter the analysis. Cases in the severe-spectrum cohort were identified via self-report, with 66.1% confirmed via chart abstraction. Of the chart abstracted cases, however, all self-reported diagnoses were correctly identified. Misclassification of a control as a case is highly unlikely, but had this occurred, it would have led to a reduction in the estimated effect. The study was limited by sample size, though post hoc power calculations indicate sufficient power to detect effect sizes larger than RR = 1.8. Thus, smaller effect sizes would not be detectable in each cohort. Borderline or null results may present a Type II error rather than a lack of effect. Further, the lack of adjustment for multiple comparisons may have resulted in spurious associations, which need to be confirmed in subsequent studies. Therefore, these results should be considered exploratory. Additionally, significant results were only noted in the combined cohorts, which may have been due to a lack of power in the stratified analyses or could be a spurious result due to the heterogeneity of the combined cohorts. Lastly, the HDP cohort was predominantly Hispanic, while the severe PE/HELLP syndrome cohort was White. The lack of racial and ethnic diversity within each cohort could limit the applicability of our results.

This study also has strengths. The case–parent dyad and triad designs enable the assessment of maternal, fetal, and parent-of-origin effects. Using log-linear models, we can account for the correlation between familial genotypes [37]. Although the study population was ethnically homogeneous within each cohort, the analysis provides insights for White and Hispanic populations and should be generalizable to any population in which these variants exist.

## 5. Conclusions

While polymorphisms and haplotypes in the *SERPINA3* gene were not associated with HDP risk, a reduction in risk was observed for maternally inherited copies of both evaluated SNPs, suggesting that genomic imprinting or maternal–fetal interaction effects may be involved. Further studies should investigate these gene associations using larger, and more diverse cohorts to reproduce these findings and improve generalizability.

## Figures and Tables

**Table 1:** (**a**) *SERPINA3* single-nucleotide polymorphism. (**b**) Pairwise linkage disequilibrium for *SERPINA3* single-nucleotide polymorphism in people with predominantly Hispanic ancestry for HDP cohort. (**c**) Pairwise linkage disequilibrium for *SERPINA3* single-nucleotide polymorphism in White group for sPE/HELLP cohort.

(**a**)									
**SNP (** **rs#** **)**	**Variant Type**	**Position**	**Consequence**	**Allele**	**Reference Allele (%)**		**Alternate Allele (%)**		**Associations**
					**LA ^1^**	**EU ^2^**	**LA**	**EU**	
rs4934	SNV	chr14:94614466 (GRCh38.p14)	SERPINA3: Missense Variant	G > A	G = 0.73	G = 0.51	A = 0.27	A = 0.48	None
rs1884082	SNV	chr14:94612340 (GRCh38.p14)	SERPINA3: 2KB Upstream Variant	G > T	G = 0.72	G = 0.49	T = 0.51	T = 0.29	Placental diseases [19]
(**b**)									
**Pairwise SNP (rs#)**	**Pairwise Linkage Disequilibrium (r2, d)**								
rs4934-rs1884082	(0.825, 1.0)								
(**c**)									
**Pairwise SNP (rs#)**	**Pairwise Linkage Disequilibrium (r2, d)**								
rs4934-rs1884082	(0.909, 1.0)								

(**a**) ^1^ Latin American population. ^2^ European population. Source: NCBI (National Center for Biotechnology Center) dbSNP (Single Nucleotide Polymorphism Database) [24]. (**b**) Source: NIH (National Institutes of Health) LDlink (Linkage Disequilibrium) tool [25] (using LDpair tool population MXL). (**c**) Source: NIH LDlink (Linkage Disequilibrium) tool [25] (using LDpair tool population).

**Table 2:** Maternal demographics stratified by case–control status.

	HDP				Severe PE/HELLP Syndrome			
Variable	*n*	Cases (*n* = 142)	*n*	Controls (*n* = 168)	*n*	Cases (*n* = 189)	*n*	Controls (*n* = 28)
Age	140	27.9 ± 7.5	168	26.9 ± 7.0	115	30.9 ± 3.9	26	32.0 ± 4.0
Ethnicity (%)								
Hispanic	136	95.8	163	96.4	-	-	-	-
Other	6	4.2	6	3.6	-	-	-	-
Race (%)								
White	-	-	-	-	189	100	28	100
Gestational age (weeks)	140	36.8 ± 3.4	168	38.7 ± 2.0	112	33.7 ± 3.8	20	39.6 ± 1.8
Pre-pregnancy weight (lbs)	140	151.0 ± 34.9	168	140.0 ± 27.2	154	144.2 ± 26.6	23	150.9 ± 23.9
Max systolic blood pressure (mmHg)	132	163.0 ± 15.8	158	118.0 ± 10.9	130	161.2 ± 23.8	-	-
Max diastolic blood pressure (mmHg)	132	97.4 ± 9.9	158	68.8 ± 9.0	130	98.6 ± 13.5	-	-
Birthweight (grams)	125	3060.0 (2395.0, 3445.0)	158	3288.0 (3018.0, 3600.0)	113	2540.1 (1360.8, 4082.3)	27	3242.3 (2041.2, 4082.3)
Nulliparity (%)	140		168		106		22	
Nulliparous		60 (42.9)		52 (31.0)		93 (87.7)		10 (45.5)
Parous		80 (57.1)		116 (69.0)		13 (12.3)		12 (54.5)
Parity (%)	138		168		106		22	
0		60 (43.5)		52 (31.0)		93 (87.7)		10 (45.5)
1		33 (23.9)		59 (35.1)		8 (7.6)		8 (36.4)
2 or more		45 (32.6)		57 (33.9)		5 (4.7)		4 (18.2)
Gravidity (%)	140		168		107		22	
1		49 (35.0)		43 (25.6)		80 (74.8)		10 (45.5)
2		34 (24.3)		48 (28.6)		19 (17.8)		6 (27.3)
3		19 (13.6)		30 (17.9)		5 (4.7)		3 (13.6)
4 or more		38 (27.1)		47 (28.0)		3 (2.8)		3 (13.6)
History of Gestational diabetes (%)	137	7 (5.1)	157	18 (11.5)	105	7 (6.7)	0	0
HELLP (%)	-	-	-	-	125	51 (40.8)	-	-
PE (%)	142	97 (68.3)	-	-	125	74 (59.2)	-	-
Superimposed PE (%)	142	3 (2.1)	-	-	-	-	-	-
Gestational hypertension (%)	142	42 (29.6)	-	-	-	-	-	-

**Table 3:** (**a**) Association between maternal and child *SERPINA3* polymorphisms and relative risk (RR) of HDPs. (**b**) Association between maternal and child *SERPINA3* polymorphisms and relative risk (RR) of severe PE/HELLP syndrome. (**c**) Combined cohort analyses: association between maternal and child *SERPINA3* polymorphisms and relative risk (RR) of HDPs or severe PE/HELLP syndrome.

(**a**)										
**SNP**	**Allele**	**Allele Frequency (%)**	**Maternal** **Single-Dose RR (95% CI)**	***p*** **-Value**	**Maternal** **Double-Dose RR (95% CI)**	***p*** **-Value**	**Child** **Single-Dose RR (95% CI)**	***p*** **-Value**	**Child** **Double-Dose RR (95% CI)**	***p*** **-Value**
rs4934	A	21.6	0.79 (0.50, 1.22)	0.28	0.97 (0.40, 2.38)	0.95	0.97 (0.63, 1.53)	0.92	1.63 (0.71, 3.82)	0.26
rs1884082	G	22.9	0.85 (0.55, 1.31)	0.45	0.85 (0.35, 2.13)	0.73	0.86 (0.55, 1.33)	0.50	1.43 (0.65, 3.12)	0.38
(**b**)										
**SNP**	**Allele**	**Allele Frequency (%)**	**Maternal** **Single-Dose RR (95% CI)**	***p*** **-Value**	**Maternal** **Double-Dose RR (95% CI)**	***p*** **-Value**	**Child** **Single-Dose RR (95% CI)**	***p*** **-Value**	**Child** **Double-Dose RR (95% CI)**	***p*** **-Value**
rs4934	A	47.4	0.76 (0.51, 1.14)	0.18	0.84 (0.49, 1.49)	0.55	1.04 (0.69, 1.61)	0.83	0.81 (0.43, 1.52)	0.52
rs1884082	T	50.3	1.10 (0.73, 1.68)	0.65	1.39 (0.78, 2.44)	0.25	1.23 (0.80, 1.90)	0.33	1.25 (0.70, 2.30)	0.46
(**c**)										
**SNP**	**Allele**	**Allele Frequency (%)**	**Maternal** **Single-Dose RR (95% CI)**	***p*** **-Value**	**Maternal** **Double-Dose RR (95% CI)**	***p*** **-Value**	**Child** **Single-Dose RR (95% CI)**	***p*** **-Value**	**Child** **Double-Dose RR (95% CI)**	***p*** **-Value**
rs4934	A	34.2	0.81 (0.60, 1.08)	0.15	1.30 (0.85, 2.03)	0.23	1.10 (0.82, 1.48)	0.51	1.54 (0.97, 2.47)	0.07
rs1884082	G	36.4	0.85 (0.64, 1.14)	0.27	1.13 (0.73, 1.74)	0.60	1.00 (0.75, 1.33)	0.99	1.41 (0.91, 2.21)	0.13

(**a**) Hardy–Weinberg Equilibrium (HWE) *p*-values: rs4924 (*p* = 0.11) and rs1884082 (*p* = 0.08). (**b**) HWE *p*-values: rs4924 (*p* = 0.43) and rs1884082 (*p* = 0.59). (**c**) HWE *p*-values: rs4924 (*p* = 0.01) and rs1884082 (*p* < 0.01).

**Table 4:** (**a**) Associations between maternal and child *SERPINA3* haplotypes and risk of HDPs (referent haplotype: T-G). (**b**) Associations between maternal and child *SERPINA3* haplotypes and risk of severe PE/HELLP syndrome. (**c**) Combined analysis of associations between maternal and child *SERPINA3* haplotypes and risk of HDPs and severe PE/HELLP syndrome.

(**a**)									
**Haplotype**	**Frequency (%)**	**Maternal** **Single-Dose RR(95% CI)**	***p*** **-Value**	**Maternal** **Double-Dose RR (95% CI)**	***p*** **-Value**	**Child** **Single-Dose RR (95% CI)**	***p*** **-Value**	**Child** **Double-Dose RR (95% CI)**	***p*** **-Value**
g-a	22.4	0.87 (0.56, 1.35)	0.53	1.03 (0.43, 2.59)	0.94	0.92 (0.59, 1.43)	0.72	1.40 (0.61, 3.15)	0.43
(**b**)									
**Haplotype**	**Frequency (%)**	**Maternal** **Single-Dose RR (95% CI)**	***p*** **-Value**	**Maternal** **Double-Dose RR (95% CI)**	***p*** **-Value**	**Child** **Single-Dose RR (95% CI)**	***p*** **-Value**	**Child** **Double-Dose RR (95% CI)**	***p*** **-Value**
g-a	51.5	0.69 (0.46, 1.05)	0.08	0.67 (0.37, 1.20)	0.18	1.09 (0.70, 1.69)	0.69	0.99 (0.54, 1.89)	0.98
(**c**)									
**Haplotype**	**Frequency (%)**	**Maternal** **Single-Dose RR (95% CI)**	***p*** **-Value**	**Maternal** **Double-Dose RR (95% CI)**	***p*** **-Value**	**Child** **Single-Dose RR (95% CI)**	***p*** **-Value**	**Child** **Double-Dose RR (95% CI)**	***p*** **-Value**
g-a	33.0	0.79 (0.59, 1.07)	0.13	1.15 (0.73, 1.82)	0.54	1.07 (0.80, 1.44)	0.64	1.58 (1, 2.52)	0.05

(**a**) HWE *p*-value = 0.08. (**b**) HWE *p*-value = 0.59. (**c**) HWE *p*-value = 0.01.

## Data Availability

The data presented in this study are available on request from the corresponding author. The data are not publicly available due to containing familial genetic information.
